# Beyond Conventional Sensing: Hybrid Plasmonic Metasurfaces and Bound States in the Continuum

**DOI:** 10.3390/nano13071261

**Published:** 2023-04-03

**Authors:** Dominic Bosomtwi, Viktoriia E. Babicheva

**Affiliations:** 1 Center for High Technology Materials, University of New Mexico, 1313 Goddard St SE, Albuquerque, NM 87106-4343, USA; 2 Electrical and Computer Engineering Department, University of New Mexico, Albuquerque, NM 87106-4343, USA

**Keywords:** Fano resonances, strong coupling, nanostructure, Rabi splitting, Kerker effect, binary arrangement, silicon nanodisks, plasmonic sensors, light-matter interactions

## Abstract

Fano resonances result from the strong coupling and interference between a broad background state and a narrow, almost discrete state, leading to the emergence of asymmetric scattering spectral profiles. Under certain conditions, Fano resonances can experience a collapse of their width due to the destructive interference of strongly coupled modes, resulting in the formation of bound states in the continuum (BIC). In such cases, the modes are simultaneously localized in the nanostructure and coexist with radiating waves, leading to an increase in the quality factor, which is virtually unlimited. In this work, we report on the design of a layered hybrid plasmonic-dielectric metasurface that facilitates strong mode coupling and the formation of BIC, resulting in resonances with a high quality factor. We demonstrate the possibility of controlling Fano resonances and tuning Rabi splitting using the nanoantenna dimensions. We also experimentally demonstrate the generalized Kerker effect in a binary arrangement of silicon nanodisks, which allows for the tuning of the collective modes and creates new photonic functionalities and improved sensing capabilities. Our findings have promising implications for developing plasmonic sensors that leverage strong light-matter interactions in hybrid metasurfaces.

## 1. Introduction

Plasmonic metasurfaces have shown great potential in sensor applications due to their ability to localize electromagnetic fields and enhance light-matter interactions. By exploiting the strong dependence of the metasurface’s optical response on its environment, plasmonic sensors based on metasurfaces can achieve high sensitivity and selectivity, making them ideal for various sensing applications, such as chemical and biological sensing, environmental monitoring, and medical diagnostics [1,2]. Moreover, the unique properties of metasurfaces, such as their tunability, polarization sensitivity, and operation in a broad spectral range, make them highly adaptable to a wide range of sensing scenarios. With ongoing advancements in nanofabrication and material design, plasmonic metasurfaces are expected to play an increasingly important role in developing next-generation plasmonic sensors with enhanced sensitivity, accuracy, and multiplexing capabilities.

Optical metasurfaces with nanoscale building blocks that support the strong coupling of the modes have generated considerable interest from researchers in recent years [3,4,5,6]. Strong mode coupling can lead to Fano resonances resulting from the mode interference and turning into asymmetric lineshapes of the scattering, absorption, reflection, or transmission spectra [3,5,6]. In essence, Fano resonances originate from the interaction between a continuum of scattered waves (bright mode) and a discrete non-radiative (dark) mode. As a fundamental resonant effect, Fano resonances have a strong response (sensitivity) to changes in the surroundings, and their sharp asymmetric spectral profiles associated with high quality factors (*Q*-factor) play a vital role in developing and implementing sensors, photonic devices, and optical applications, including filters, modulators, lasers, optical switches, and broadband reflectors [3,4,5,6,7].

Previously, Fano resonances have been explored in various structures, including dielectric and plasmonic nanostructures, photonic crystals, and optical metamaterials [5,6,7,8,9,10]. Hybrid plasmonic-dielectric nanostructures are highly useful, as they allow for the utilization of effects related to both plasmonics and high refractive index materials, enabling new opportunities in areas such as sensing, imaging, and energy conversion. Plasmonic field enhancement occurs at the interface of metal and dielectric when the interaction between the optical wave and the collective oscillations of free electrons in the metal, known as plasmons, leads to a higher electric field in the dielectric region [3,5,6,11,12,13]. On the other hand, Mie resonances occur in high-refractive-index nanoparticles when incident electromagnetic waves excite the nanoparticles to oscillate at their resonant frequencies, resulting in the scattering and absorption of light [7,14,15,16]. Zhao et al. have designed all-dielectric binary silicon nanodisk arrays supporting the excitations of Fano resonances [7]. High *Q*-factors of Fano resonances in optical metastructures composed of asymmetric double bars have been demonstrated by Moritake et al. [9], and Khanikev et al. have discussed the applications of Fano-resonant metamaterials in sensing, switching, lasing, and nonlinear optics, as well as their potential use in developing efficient solar cells and enhancing the performance of optoelectronic devices [10]. Furthermore, Byrne et al. have also investigated the origin of Fano resonances in photonic crystal slabs [17], and Luk’yanchuk et al. have studied Fano resonances in plasmonic structures and metastructures [18]. Binary nanoparticle arrays generate a large number of modes resulting in Fano resonances due to the strong interaction between the plasmonic resonances of nanoparticles with different sizes and positions with respect to each other [19].

Recently, the physics of bound states in the continuum (BICs) has become the subject of great importance to researchers due to the possibility of near-perfectly confined optical energy at the nanoscale and, at the same time, coupling to the external illuminating/radiating waves [14,20,21,22,23,24,25,26,27,28,29]. This unique behavior is not found in traditional cavity systems with resonators, photonic bandgaps, total internal reflection, reflective mirrors, or others, where the waves are prohibited from radiating into the surrounding medium but remain trapped locally under constructive/destructive interference conditions [20,21]. Bound states in the continuum arise due to the strong coupling between a discrete state with a continuum state under the conditions of destructive interference leading to the collapse of the width of the Fano lineshape (bandgap), resulting in resonances with a very high, almost infinite *Q*-factor. However, an ideal BIC excitation in a photonic structure is a theoretical concept that requires an infinite *Q*-factor or at least one infinite dimension of the structure, leading to the complete disappearance of the Fano lineshape width. Thus, due to fabrication imperfections and material absorption in real life, the width of Fano resonances and *Q*-factor become finite at the BIC point, and a true BIC transforms into a so-called *quasi-BIC* [20,21,22].

Bound states in the continuum and associated optical processes, such as Fano resonances and Rabi splitting, have been applied in designing metasurfaces and photonic elements, such as waveguides, coupled resonators, and dielectric gratings [21,22,23,24,25]. Bound states in the continuum have potential applications in various areas, such as sensing, optical filtering, laser technology, nonlinear photonics, and coherent light generation [23,24,25,29]. Since the 1980s, strong mode coupling leading to their splitting (so-called Rabi splitting) in the spectra of atomic and quantum systems has attracted great attention from researchers, and this phenomenon has gained increasing interest in nanophotonics in recent years [30,31,32,33,34,35,36]. Initially proposed and observed in atomic and molecular systems, Rabi splitting occurs in a strong coupling regime where the energy frequently oscillates between a single photon and a two-level quantum emitter leading to the splitting of the spectra of the scattering parameters of the quantum emitter in a high *Q*-factor cavity [30,31,32,33]. Therefore, Rabi splitting occurs in the realm of strong coupling where the coherent energy exchange rate between the emitter and matter exceeds its energy dissipation rate, which causes anti-crossing in the dispersion relation of the system. Thought to be a fundamentally pure quantum mechanical effect, Rabi splitting has been investigated and observed in various quantum systems, such as quantum-dot microcavity, emitter-cavity systems, and recently in classical and optical systems, such as waveguides, plasmonic nanoparticles, photonic crystals, and periodic structures [30,31,32,35,36]. Strong coupling in quantum and classical systems is very important in many practical applications, such as quantum information processing, superconducting resonators, and optical detection [31,32,33,34,35,36].

**Figure 1:**
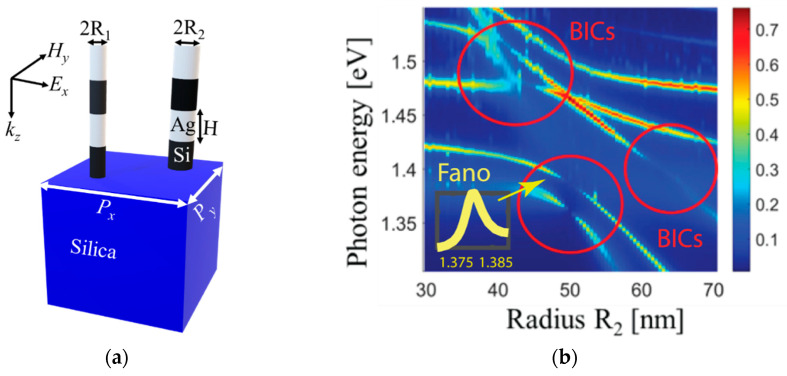

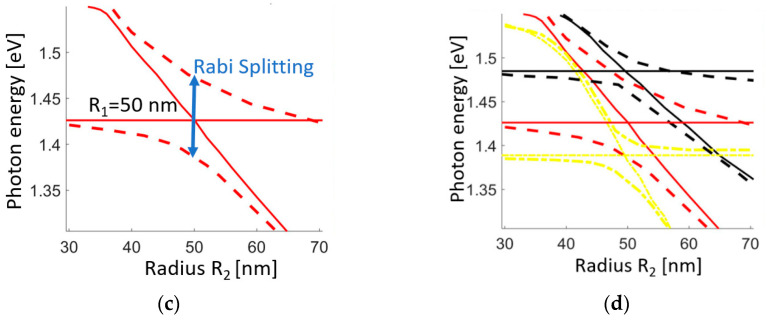
(**a**) Schematic of the proposed hybrid metasurface under consideration, where the unit cell consists of two elements (nanopillars). Each element has four nanodisks: two plasmonic made of silver and two made of high-refractive-index material, silicon. The nanodisks are of height *H =* 120 nm and radii *R*_1_ = 50 nm (fixed) and *R*_2_, varied from 30 to 70 nm. The pairs are arranged in periodic array with periods *P_x_* = *P_y_* = *P* = 550 nm. The array is illuminated with the *x*-polarized light at the normal angle. The substrate and superstrate materials are silica. Considering the center of the unit cell has coordinates (0, 0), the first element has coordinates (−*P_x_*/4, −*P_y_*/4), and the second element has coordinates (*P_x_*/4, *P_y_*/4). (**b**) Numerical results and mode contours are for modeling the proposed nanostructure: The simulated absorption spectra versus radius of nanoantenna showing sextuple Fano resonances and triple Rabi splitting. It shows quintuple BICs in the nanostructure. Bound states in the continuum are shown in red circles due to the collapse of the widths of Fano resonances observed in the nanostructure. Inset: Fano profile of the mode at ~1.38 eV for *R*_2_ = 48 nm. (**c**) Contours of one mode pair. The horizontal line corresponds to the mode in Element #1 (unchanged due to the constant *R*_1_ = 50 nm), and the diagonal line corresponds to the mode in Element #2 that changes along with *R*_2_. The solid lines are the results for a single element in the unit cell, while the dashed lines are the results of modeling two elements. (**d**) Contours of three pairs of the nanostructure modes.

In this work, we report on a novel metasurface design of a layered hybrid plasmonic-dielectric metasurface that enables robust mode coupling and the emergence of BIC, leading to resonances with a high quality factor. We study the scattering response of multilayer silver-silicon nanodisks arranged in pairs in a periodic lattice (Figure 1a). This nanostructure supports Fano resonances, which can be tuned to observe Rabi splitting of the modes and their cancellation in a BIC point. By fabricating and experimentally characterizing silicon nanodisks arranged in a binary periodic array, we demonstrate the practical observation of Fano resonances and the generalized Kerker effect. We illustrate the excitation of Fano resonances in the metasurface, which originate from the coupling of resonant dark and bright modes of the multiple boundaries of metal and dielectric. We discuss the existence of Rabi splitting as a result of the strong interaction between the nanostructure modes due to the constructive coupling between the resonant dark and bright modes in the nanostructure. We show strong mode confinement in the metasurface elements resulting in multiple quasi-BIC points due to destructive interference and the collapse of the width of the Fano lineshape. This BIC-based hybrid metasurface is highly sensitive and selective because a small parameter mismatch in a binary array can create a BIC within the bandgap of the coupled modes. The enhanced light absorption and/or scattering associated with the Kerker condition can lead to stronger and more selective sensing signals in plasmonic sensors by tuning the geometric and material parameters of nanoparticle arrays, potentially enabling the highly specific and sensitive detection of analytes in a range of sensing platforms.

## 2. Coupled Modes and Bound States in the Continuum

To start with, we consider a hybrid plasmonic metasurface consisting of a nanodisk array (Figure 1a). This is a binary array as the unit cell includes two elements with a slight mismatch in their parameters. The metal and dielectric structures are made of silver (Ag) and silicon (Si), and the metasurface is positioned on a silicon oxide substrate with the same superstrate surrounding. The length of the array unit along the *x-* and *y*-directions is *P_x_* = *P_y_* = 550 nm. Each nanodisk in the unit cell has the same height *H =* 120 nm, but they differ in radii: the first element has the radius *R*_1_*,* and the second has *R*_2_. Numerical simulations are carried out using the CST Studio Suite software package. The periodic boundary conditions are set in the *x*- and *y*-directions to ensure that the simulation accurately represents a repeating system in those dimensions. In other words, the simulation “wraps around” in the *x*- and *y*-directions to create an infinite lattice structure. Open boundary conditions are used in the *z*-direction, which is the direction of wave propagation, to allow waves to travel freely in and out of the simulated region. This enables the accurate modeling of wave behavior and avoids any artificial reflections or interference caused by closed boundaries in the *z*-direction. The metasurface is illuminated by a plane wave polarized in the *x*-direction (electric field *E* along the *x*-axis) at normal incidence. Section 5.1 provides further details on the numerical simulation.

In the context of the optical properties of an array or cluster of nanoparticles, a “mode” (or “state”) refers to a specific type of oscillation or vibration that can occur within the system. Each mode has a particular frequency associated with it, and the system can exhibit multiple modes depending on its geometry, size, and composition. On the other hand, a “resonance” refers to the specific frequency at which the system exhibits a strong response to an external excitation (such as light). When the excitation frequency matches the mode frequency of the system, the system can absorb or scatter the light very efficiently, resulting in a resonance peak in the system’s optical response. Resonances can be broad or narrow, depending on the system’s damping properties and the coupling between the modes. Thus, modes are inherent properties of the nanoparticle system, while resonances result from the interaction between the system and external stimuli, such as light.

Fano resonances are characterized by a sharp peak asymmetrically embedded in a broader feature, which can be observed in the reflection, transmission, or absorption spectra. One way to identify a Fano resonance in the spectrum of the metasurface is to analyze the asymmetry of the spectral line shape. Another way to identify Fano resonances is to look for the presence of a phase change in the transmitted or reflected light, which occurs due to the interference between a discrete mode and a continuum of modes. Similarly, to identify a spectral feature close to a BIC point in the metasurface, one can analyze the linewidth of the resonance. At the BIC point, the linewidth of the resonance becomes very narrow due to the destructive interference between the discrete and continuum modes. In general, the analysis of the electromagnetic field intensity and polarization patterns near the resonance can also provide useful information to identify the spectral feature close to the BIC point.

To analyze the various mode excitations in the metasurface, we calculate light absorption across the entire nanostructure. By identifying the spectral points with the greatest power loss, we can pinpoint the specific nanostructure modes that are present. When identifying closely excited resonances in arrays, it is important to avoid using characteristics such as reflection and transmission, as these scattering quantities can be influenced by interference and lead to misleading results. Figure 1b presents the absorption spectra of a proposed structure with two elements in the unit cell. One element has a fixed *R*_1_ value of 50 nm, while the other has *R*_2_ values that range from 30 nm to 70 nm. By manipulating individual array element radii while keeping another constant, we can observe Fano resonances and induced Rabi splitting within the nanostructure. Figure 1b showcases several Fano resonances that arise from the overlap between hybridized modes consisting of both bright (radiative) and dark (non-radiative) modes due to the lattice coupling of their collective modes.

**Figure 2:**
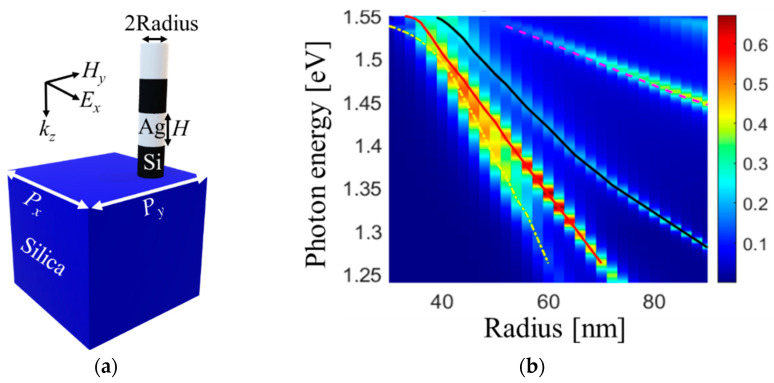
Nanostructure with one element. (**a**) Schematics of a single element in the unit cell. The element radius is *R*. Other geometrical parameters, materials, and illumination are the same as in the binary array. (**b**) Absorption for different radii. We perform calculations for a single element to aid in analyzing mode excitations in the binary array. Dot-dash yellow, solid red, and solid black lines are eyeball fit. They are subsequently transferred to Figure 1c,d to interpret the absorption mode maps.

Because elements are arranged in the periodic array, the metasurface supports collective resonances. Collective resonances are excited in periodic arrays, and their properties are strongly influenced by the periodicity of the array [37,38,39,40]. To better understand the nature of mode excitation, we utilize a nanostructure with only one element in the array’s unit cell, as shown in Figure 2. By varying the element radius *R* and tracing the resulting modes that manifest as peaks in the absorption spectra, we are able to identify a consistent trend. Using a visual eyeball fit, we draw lines to represent the excited modes, with dot-dash yellow, solid red, and solid black lines indicating a nearly linear change in the mode position with changes in the element radius. These lines correspond to the solid diagonal lines in the figures for a binary array (Figure 1c,d), while the solid horizontal lines correspond to the case of a fixed radius *R*_1_ of 50 nm. Thus, the diagonal and horizontal solid lines in Figure 1c,d represent the results of the calculations for an array with only one element in the unit cell.

We performed a similar analysis of the absorption maxima for a binary array, akin to the case of a unit cell with only one element. Using an eyeball fit, we identified the excited modes, which are represented by dashed yellow, red, and black lines in Figure 1d. A key observation is an excellent agreement between the results obtained for a unit cell with only one element and those obtained for the binary array, which highlights the robustness of our analysis method. While the solid and dashed lines in Figure 1d come from two different types of simulations, we see that the dashed lines are the modes experiencing Rabi splitting because of the binary nature of the array. This observation is significant as it highlights the effects of lattice coupling on mode excitation and behavior.

Figure 1c,d show mode energy maps with one mode pair only and three mode pairs, respectively. One can observe mode anticrossing as a result of the Rabi splitting due to the coupling of nanostructure modes. For instance, in Figure 1c, the Rabi splitting energy (shown in blue double-head arrows) is due to the strong coupling between nanostructure modes. One can observe triple anticrossings indicated in the dashed lines due to the splitting of the resonant modes in the nanostructure as a result of the strong coupling between different Fano resonances. Figure 1d illustrates three anticrossings in yellow, red, and black lines corresponding to triple Rabi splittings with energies 0.055, 0.08, and 0.025 eV, respectively.

In Figure 1b, one can see about five discontinuities in the nanostructure modes in the vicinity of the anticrossings in the absorption colormap shown in three red circles. These discontinuities occur when radiative losses in the nanostructure are suppressed due to destructive interference and BIC formation. These discontinuities indicate singularities in the Fano parameter that lead to the collapse of the Fano lineshapes (bandgap), resulting in the trapping of electromagnetic modes in the nanostructure, with the *Q*-factor increasing rapidly and eventually reaching a maximum value due to finite size effects [34]. This rapid growth of the *Q*-factor is attributable to BICs, where strongly coupled modes in the nanostructure undergo destructive interference in the surrounding medium of the nanostructure.

Our results suggest that even a small deviation from matching resonance positions, which are determined by the element dimensions, can result in Rabi splitting. This is evident through shifts in peak positions and characteristic asymmetrical spectral profiles. This hybrid metasurface is a layered plasmonic-dielectric structure that supports high-quality resonances via mode coupling and BIC emergence. Tunable Fano resonances enable observation of Rabi splitting and mode cancellation at BIC points. The strong interaction between resonant dark and bright modes leads to Rabi splitting, while strong mode confinement in the elements generates multiple quasi-BIC points through destructive interference and Fano lineshape narrowing.

In binary nanoparticle arrays, BICs can be achieved by tailoring the geometric parameters of the individual elements to create destructive interference between modes, leading to unique optical properties with potential applications in sensing. By introducing a small parameter mismatch in a binary array constituting the metasurface, a BIC can be created within the bandgap of the coupled modes. This BIC can then be used to enhance the light-matter interaction within the metasurface region, resulting in highly sensitive and selective sensing. The presence of the BIC also leads to a sharp resonant peak in the transmission or reflection spectrum, making it easy to detect changes in the surrounding environment that perturb the BIC, such as the adsorption of a biomolecule or the presence of a gas. Additionally, BIC-based plasmonic sensors are highly robust against fabrication imperfections and environmental changes due to the highly localized nature of the BIC. This allows for highly reproducible sensing with minimal drift over time.

## 3. Kerker Effect and Sensitivity

In the previous section, we demonstrated the strong coupling of hybrid modes and BIC points emerging from their interaction. In this section, we show the effects of the interference between these modes, which leads to suppressed reflection because of the generalized Kerker effect and enhanced sensitivity of this hybrid metasurface due to these abrupt changes in reflection.

**Figure 3:**
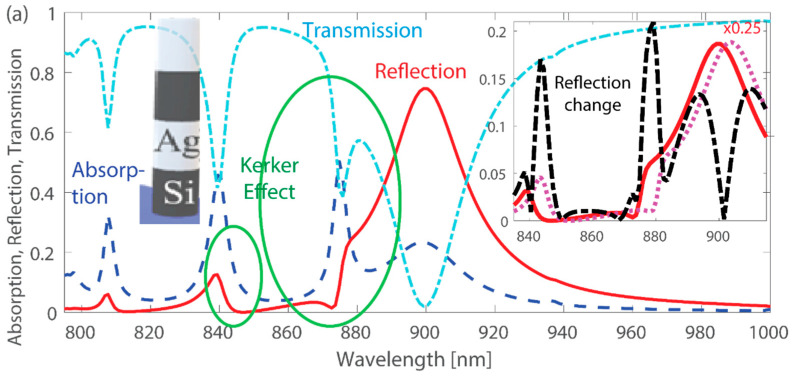

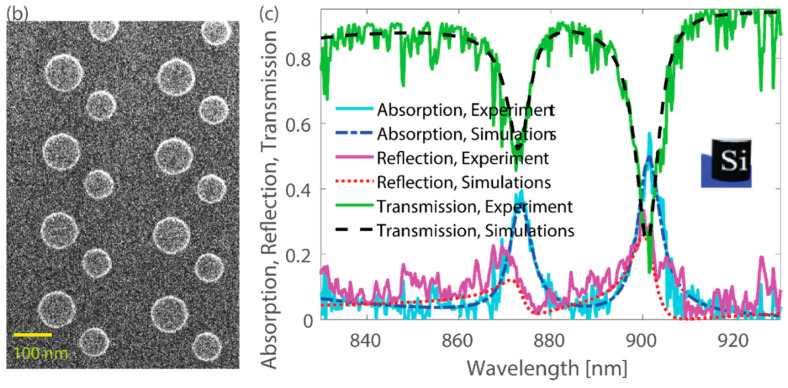
Multiple mode excitations and generalized Kerker effect. (**a**) Absorption, reflection, and transmission for a binary array of the hybrid metasurface. The nanodisks are of height *H =* 120 nm and radii *R*_1_ = 50 nm and *R*_2_ = 30 nm, and the pairs are arranged in the periodic array with periods *P_x_* = *P_y_* = *P* = 550 nm. The two green circles highlight regions where the generalized Kerker effect is observed (suppressed reflection due to scattering compensation between multiple resonances). Inset: Changes in the reflection spectra when the surrounding refractive index is increased by 0.02. The dot-dash black lines indicate the absolute change, while the solid red and dashed magenta lines show the reflection scaled by 0.25 for visual clarity. Notably, the largest absolute change in reflection occurs at spectral points corresponding to the generalized Kerker effect, with values reaching up to 0.2. (**b**) Scanning electron microscope image of binary silicon array. (**c**) Absorption, reflection, and transmission for a binary silicon array with one layer of silicon nanodisks of height *H =* 140 nm and radii *R*_1_ = 100 nm and *R*_2_ = 70 nm, and the pairs are arranged in the periodic array with periods *P_x_* = 550 nm and *P_y_* = 380 nm. Insets: Schematics of the elements constituting the metasurface (hybrid in (**a**) and silicon in (**c**)).

We plot reflection and absorption for the case of *R*_1_ = 50 nm and *R*_2_ = 30 nm (Figure 3a). We observe that there are specific spectral regions where the mode excitations are strong, and the metasurface supports multiple resonances manifested as absorption peaks. At the same time, in some of these regions, reflection is significantly suppressed. This phenomenon occurs because the scattering from multiple modes destructively interferes with each other in the direction of reflection, similar to the concept of backward scattering in finite-size nanostructures. This effect is commonly known as the generalized Kerker effect. While the traditional Kerker effect arises from the scattering compensation between electric and magnetic dipoles, the involvement of other multipoles in the process can lead to the compensation of scattering from higher-order multipoles, known as the generalized Kerker effect.

The Kerker condition corresponds to a specific set of geometric and material parameters that lead to the suppression of forward scattering and the enhancement of backward scattering in nanoparticle systems [37,39]. At the point where the Kerker condition is satisfied, the reflection spectra exhibit a sharp, asymmetric peak that is associated with strong near-field coupling and resonant dipole-dipole interactions among the nanoparticles. This unique spectral response has significant implications for the design of optical sensors as well as photonic devices and applications, such as efficient light harvesting in solar cells and ultrasensitive biosensors.

The generalized Kerker effect not only leads to a suppression of reflection, but also causes rapid and significant changes in reflection values. This abrupt change can be utilized in sensing applications because of the pronounced response to the change of system parameters, e.g., refractive index or dimensions. Our proposed hybrid metasurface exhibits a high sensitivity to changes in the refractive index of the surrounding medium, with alterations as small as 0.02 resulting in significant changes in reflection, up to a maximum of 0.2 (see inset in Figure 3a). In the numerical simulations, the refractive index of the medium surrounding the binary plasmonic-dielectric elements is altered by 0.02 as a change from 1.45 to 1.47. Most importantly, the largest absolute change in reflection occurred at spectral points corresponding to the generalized Kerker effect, with values reaching up to 0.2 (at the wavelength of approximately 843 and 879 nm). These results suggest that the abrupt changes in reflection from the hybrid metasurface can be exploited to achieve an enhanced response and higher sensitivity to changes in the surrounding environment, making it a promising platform for sensing applications.

Plasmonic nanostructures have shown great potential for sensing applications due to their ability to confine light into nanoscale volumes, leading to high sensitivity and selectivity [41,42,43,44,45,46]. The spectral tunability of plasmonic nanostructures is limited by the intrinsic properties of the plasmonic materials and the geometrical design of the nanostructure. The plasmonic resonance frequency of a metal nanostructure is determined by the metal permittivity and the size and shape of the nanostructure. While the size and shape of the nanostructure can be tailored to some extent, the intrinsic properties of the metal cannot be easily altered, and this limits the tunability of the plasmonic resonance frequency.

The proposed hybrid metasurface offers a higher degree of tunability in their resonant frequency compared to plasmonic nanostructures. This is because the hybrid metasurface can be designed to have a broad range of effective responses by controlling the geometry and arrangement of the constituent elements. This allows for a wider range of tunability in the resonance frequency and makes the hybrid metasurface more versatile for various sensing and imaging applications.

The sensing performance of metasurfaces can be analyzed through various mechanisms. The traditional sensing mechanism is based on detecting the shift in the resonance wavelength, which changes as the refractive index of the environment varies. However, there are other sensing mechanisms based on the change in the resonance amplitude or phase, and these mechanisms can be more sensitive in certain situations. In the case of refractive index sensing, we observed a wavelength shift of approximately 5 nm for a change in the refractive index of 0.02, which corresponds to a sensitivity of approximately 250 nm/RIU. This value is slightly higher than those reported recently for dielectric nanostructures [47,48], indicating the potential of a hybrid metasurface for high-sensitivity sensing. Additionally, this value is within the range of values reported for plasmonic nanostructures [49], which are known for their high sensitivity and limited spectral tunability. Therefore, the hybrid metasurface offers a promising alternative for high-performance sensing applications with enhanced tunability.

To demonstrate the generalized Kerker effect, we fabricate larger silicon nanodisks than previously discussed (*R*_1_ = 100 nm and *R*_2_ = 70 nm, Figure 3b) and optically measure the reflection from this silicon binary array (Figure 3c). Details about the nanofabrication and characterization procedures are provided in the Section 5. The binary periodic array consists of pairs of nanodisks with a thickness of *H* = 140 nm, arranged with periods *P_x_* = 550 nm and *P_y_* = 380 nm. This nanostructure differs from the one presented above as it has only one silicon layer and can be fabricated using well-established procedures. The absence of additional metal-silicon interfaces reduces the number of resonances supported by the metasurface. Two resonances are observed in both simulations and measurements, with good agreement between them. The observed discrepancy can be attributed to factors such as oblique incident light components and nanodisk imperfections.

Similar to the case of a hybrid metasurface, we observe that the silicon nanodisks exhibit strong mode excitations in certain spectral regions, resulting in multiple resonances and absorption peaks. The measured response simultaneously exhibits significantly suppressed reflection due to the destructive interference among the backward scattered waves (at the wavelength of approximately 908 nm and 877 nm), which is referred to as the generalized Kerker effect. Overall, our results demonstrate the practical observation of resonances in a binary array.

## 4. Bright and Dark Modes

The paired elements with different radii and resonant frequencies exhibit corresponding collective modes at different spectral points. Through hybridization, these collective lattice modes result in resonances that are either in- or out-of-phase with respect to each other or the incident wave [7,50,51]. The out-of-phase field configuration is responsible for forming a subradiant (dark) mode, while the in-phase field configuration is accountable for forming the superradiant (bright) mode. In the case of the out-of-phase field configuration, the electric fields in the two elements oscillate in opposite directions. Therefore, by examining the near-field interactions, we observe that the dark mode excitation represents a null net dipole moment (two elements cancel each other’s dipole moment) with an extended or prolonged lifetime. The radiated fields from the two elements interfere destructively in the far-field zone and do not couple to free space radiation. Consequently, the subradiant (dark) mode cannot escape from the locality of the metasurface and can be seen as a “trapped” mode. On the other hand, in the case of the in-phase collective mode, the two elements oscillate in the same or opposite direction with the incident light wave. Hence, the radiated fields from the two elements interfere constructively in the far-field zone and form a superradiant (bright) mode [50,51].

**Figure 4:**
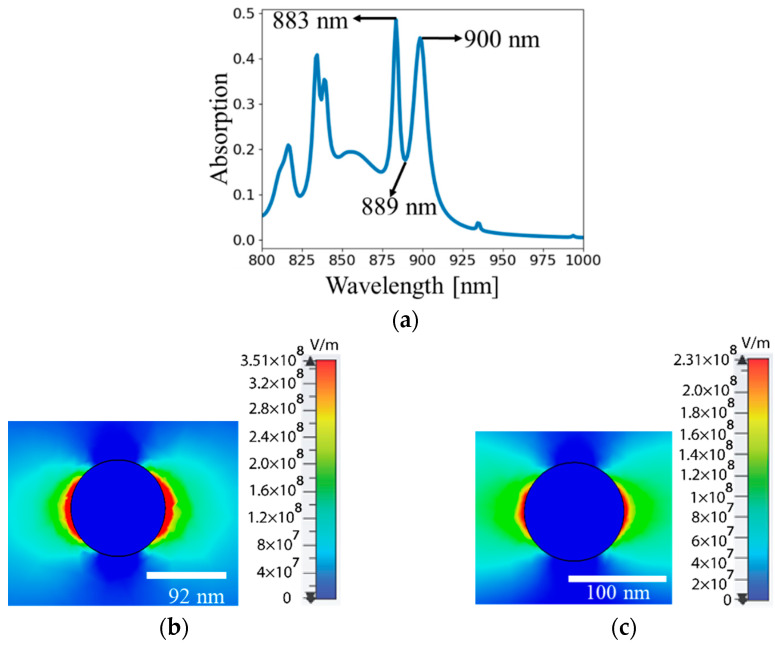

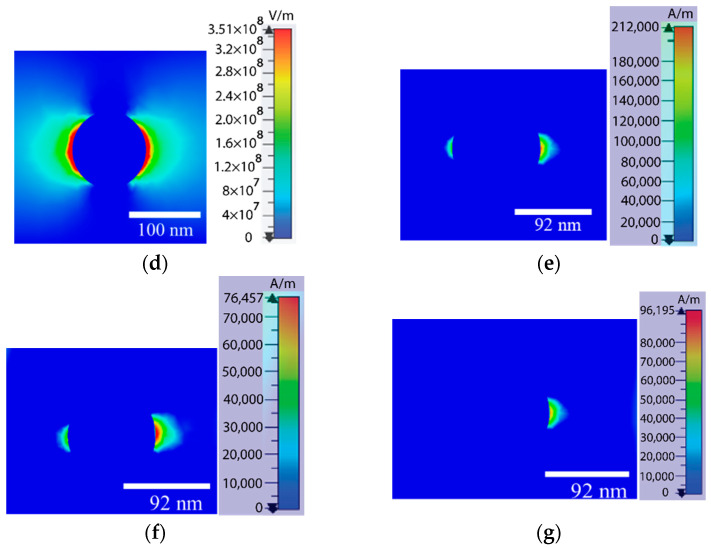
Electric (*E_x_*) and magnetic (*H_y_*) field distributions outside the nanodisks with radii *R*_1_ = 50 nm and *R*_2_ = 46 nm. (**a**) Absorption spectra of nanoantenna with radius *R*_2_ = 46 nm. (**b**) *E_x_* at λ = 900 nm, *z =* 240 nm, in element #2. (**c**) *E_x_* at λ = 883 nm, *z* = 240 nm, in element #1. (**d**) *E_x_* at λ = 889 nm, *z =* 240 nm, in element #1. (**e**) *H_y_* at λ = 889 nm, *z* = 240 nm, in element #2. (**f**) *H_y_* at λ = 900 nm, *z* = 240 nm, in element #2. (**g**) *H_y_* at λ = 883 nm, *z* = 240 nm, in element #2.

The overlap of the out-of-phase and in-phase collective modes brings about the sharp asymmetric Fano lineshapes in the wavelength-dependent characteristics of the scattering parameters of the array lattice at excitation wavelengths in the proximity of Rayleigh anomalies [11,52]. To understand the formation of Fano resonances, we identify the subradiant and superradiant modes associated with Fano resonances in the absorption spectrum shown in Figure 4a for the binary array with radii *R*_1_ = 50 nm and *R*_2_ = 46 nm. In Figure 4b–g, we illustrate the electromagnetic field distributions in the cross-section of the nanodisks in order to understand the Fano-resonance features shown in Figure 1. We plot the *x*-component of the electric field (*E_x_*) distribution of the unit cell of the two particles in the array across the centers of the nanoantennas (*z* = 240 nm), as shown in Figure 4. First, we analyze the electric field distributions at wavelengths of 900 nm and 883 nm around the nanoantennas of radii *R*_2_ = 46 nm and *R*_1_ = 50 nm. Figure 4b,c show bright (superradiant) modes as a result of the constructive interference of the radiated fields from the two nanoantennas in the far field. Figure 4d illustrates the electric field distribution at a wavelength of 889 nm as a result of the excitation of the out-of-phase field configuration, where the particles oscillate in opposite directions. The radiated fields in the far field cancel each other out as a result of the destructive interference leading to the formation of dark (subradiant) modes, and hence a significant reduction in the absorption. As we discussed above, the overlap of the superradiant and subradiant modes results in the formation of a sharp asymmetric Fano lineshape in the spectral absorption of the nanoantenna array. In Figure 4e–g, we demonstrate the distributions of the *y*-components of the magnetic field at wavelengths of 889 nm, 900 nm, and 883 nm. We see that the field remains unperturbed in this type of binary array, and it agrees well with other observations that these nanostructures are typically associated with a weak magnetic response [7].

## 5. Method

### 5.1. Numerical Simulations

To perform full-wave simulations, we used a frequency-domain solver available in the CST Studio Suite commercial package. In our simulations, the incident wave’s electric and magnetic fields were oriented along the *x*- and *y*-directions, respectively. To account for the decay of near fields around hybrid elements, we included a substantial distance between the metasurface and domain boundaries. When considering diffractive effects, it is crucial to note that the required distance between a nanoparticle array and boundaries is significantly greater than that of a single nanoantenna. In our simulations, we use the Floquet-Bloch boundary conditions in the *x*- and *y*-directions, while open boundaries are applied in the *z*-direction. We account for the first 18 modes to ensure the complete attenuation of any incoming waves originating from the nanoantenna scattering into different diffraction orders. This attenuation is achieved through open boundaries.

### 5.2. Nanofabrication

The fused silica sample was clean and coated with 10 nm sapphire deposited with an e-beam evaporator (KJL Custom 9 kV) at a 0.5 Å/s rate. Next, silicon was sputtered (KJL PVD-75) to a total thickness of 140 nm. The sample was then spin-coated with PMMA 495-A2 (4000 rpm) and baked at 180 °C for 3 min. The same was repeated with PMMA 950-A2. Electron-beam exposure was done with JEOL JBX 6300-FS, 60 μm aperture, 100 keV energy, and 1 nA current, and the sample was developed in MIBK-IPA 1:3 solution for 90 s. Chromium was deposited with an e-beam evaporator (CVC Custom 10 kV) at a 0.4 Å/s rate to a total thickness of 20 nm. The sample was immersed in Remover PG and heated to 78 °C for 2 h and 30 min. Next, we removed the e-beam resist by thoroughly rinsing it with acetone. To etch the silicon underneath the chromium hard mask, we used the ICP SiDRIE Bosch Etch system (Modified Plasmatherm SLR-770) at 5 °C. The total etch time was approximately 75 s. We used a thin sapphire layer as a stop layer between the fused silica substrate and the silicon to prevent etching from damaging the substrate or destroying the silicon that is part of the fused silica. The chromium layer was removed using CR7 chromium etchant for 1.5 min, followed by a 5-min piranha clean using a 3:1 mixture of H_2_SO_4_ and H_2_O_2_ (e.g., 9 mL of Sulfuric Acid A300-500 and 3 mL of Hydrogen Peroxide). The sample was cleaned with Down Stream Plasma Ash (PT Batchtop with Low-Frequency Plasma Source) for 10 min. Finally, to realize the index-matching environment on top of the nanodisks, we spin-coated multiple layers of thick PMMA, as follows. The sample was spin-coated with HMDS at 2000 rpm to improve polymer adhesion, followed by spin-coating with PMMA 950-A9 at 5000 rpm. The PMMA layer was then baked at 180 °C for 1 min, and this spin-coating step was repeated two more times. However, the last cycle was performed at 3000 rpm since the smoothness of the final layer was deemed less critical. As a result, a polymer layer of approximately 4 μm in thickness was formed on top of the nanodisks.

### 5.3. Optical Measurements

We measured the reflection and transmission of the sample using a custom setup connected to the ASEQ Instruments LR1 spectrometer operating in the range of 200–1200 nm, with a resolution of approximately 2 nm. Broadband light was directed through a series of optical elements and lenses to focus on a sample spot of approximately 100 μm in beam waist, while a small aperture placed between the last lens and the sample allowed only light normal to the sample with no more than a couple of degrees deviation. To obtain accurate results, we normalized the reflection from the array to the reflection from the mirror with the same thickness positioned on the same stage as the sample during the measurement. Similarly, we normalized the transmission through the array to the transmission through the sample spot without the nanostructure. The absorption spectrum was calculated by subtracting the normalized reflection and transmission values from the unit.

## 6. Conclusions

We demonstrated a hybrid metasurface that can be applied in designing BIC-based plasmonic sensors to achieve highly sensitive and selective detection for a wide range of applications. Through the numerical investigation of a hybrid plasmonic metasurface, we have analyzed the strong coupling between the modes induced in the binary array by periodic arrangement. Our study has involved fabricating silicon nanodisks arranged in a binary periodic array, enabling us to observe Fano resonances and the generalized Kerker effect in a practical setting. Through optical characterization, we have been able to confirm the resonant behavior of the nanodisks and establish their potential for applications in sensing as well as a variety of optical and photonic devices.

Our results demonstrate that the excitation of collective modes enables strong energy confinement at the subwavelength scale. The hybrid plasmonic metasurfaces exhibit a tunable resonant response, and by altering the size of one of the elements in the binary metasurface, we observed three related optical phenomena: Fano resonances, bound states in the continuum (BICs), and Rabi splitting. Our findings indicate that even a slight mismatch in the resonance positions controlled by the element dimensions can lead to Rabi splitting, which was observed as changes in peak locations and distinct spectral asymmetries. These hybrid elements can be utilized in designing directional nanoantennas and their lattices that are optimized for use in flat optical applications, such as metastructures and transdimensional photonic lattices. Our work highlights the potential of hybrid plasmonic metasurfaces with multi-segment elements in enabling strong light-matter interactions at the nanoscale, providing a valuable framework for designing multi-resonant metasurfaces operating in the visible and near-infrared regimes.

We have shown that the enhanced scattering associated with the Kerker condition can lead to stronger and more selective sensing signals in plasmonic sensors, as the backward scattering direction is typically more sensitive to changes in the surrounding environment. By tuning the geometric and material parameters of plasmonic nanoparticle arrays to satisfy the Kerker condition, one can achieve a highly specific and sensitive detection of molecular and biomolecular analytes. This approach can potentially be implemented in various plasmonic sensing platforms, including those based on localized surface plasmon resonance and collective lattice effects, with potential applications in biomedical diagnostics, environmental monitoring, and food safety testing.

## Data Availability

The data presented in this study are available on request from the corresponding author. The data are not publicly available due to technical, resource, and time constraints.
